# Do Cooking Classes for Nutrition Students Improve Their Eating Competence and Cooking Skills? A 1-Year Follow-Up in a Sample of Brazilian Public University Students

**DOI:** 10.3390/nu18020259

**Published:** 2026-01-14

**Authors:** Julyana Nogueira Firme, Renata Puppin Zandonadi, Millena Amaral Santana, Rafaella Dusi, Eduardo Yoshio Nakano, Fabiana Lopes Nalon de Queiroz, Luanna Ortiz Costa Ribeiro, António Raposo, Zayed D. Alsharari, Raquel B. A. Botelho

**Affiliations:** 1 Department of Nutrition, Faculty of Health Sciences, Campus Universitário Darcy Ribeiro, University of Brasília, Brasilia 70910-900, Brazil; julyanafirme@gmail.com (J.N.F.); nutri.millenaamaral@gmail.com (M.A.S.); rafaella.dusi@gmail.com (R.D.); fabinalon@hotmail.com (F.L.N.d.Q.); luanna.ocr@gmail.com (L.O.C.R.); raquelbotelho@unb.br (R.B.A.B.); 2 Department of Statistics, University of Brasilia, Brasilia 70910-900, Brazil; nakano@unb.br; 3 CBIOS (Research Center for Biosciences and Health Technologies), ECTS (School of Health Sciences and Technologies), Lusófona University, Campo Grande 376, 1749-024 Lisboa, Portugal; 4 Department of Health Rehabilitation Sciences, Faculty of Applied Medical Sciences, University of Tabuk, P.O. Box 741, Tabuk 71491, Saudi Arabia; zalsharari@ut.edu.sa

**Keywords:** cooking classes, cooking skills, eating competence

## Abstract

**Background:** The decline in traditional cooking practices and the increased consumption of ready-to-eat meals have raised concerns about dietary quality and health, especially among university students. Nutrition students, despite their academic training, often struggle to translate theoretical knowledge into healthy eating practices. Culinary classes in academic settings have emerged as promising strategies to enhance both cooking skills (CS) and eating competence (EC). **Objectives:** This study aimed to evaluate the impact of a 12-month cooking class program on the development of culinary skills and eating competence among nutrition students at a public university in Brazil. **Methods:** A longitudinal study was conducted with 42 nutrition students who completed a structured questionnaire at three time points: baseline, after 6 months, and after 1 year of participation in sequential cooking-related subjects. Data were collected using the Brazilian Cooking Skills and Healthy Eating Questionnaire (QBHC) and the Brazilian version of the Satter Eating Competence Inventory (ecSI2.0™BR). Statistical analyses included a repeated-measures ANOVA and a Pearson correlation. Bonferroni post hoc comparisons were conducted following the repeated-measures ANOVA to identify the time points at which significant differences occurred. **Results:** Participants, predominantly young females (78.6%, mean age 21.07 ± 2.71 years), demonstrated high CS at baseline and showed significant improvements over time (*p* < 0.05). At baseline, 59.5% of participants (*n* = 25) were considered competent eaters (EC ≥ 32). Knowledge in cooking terms and techniques increased after one year (*p* = 0.023). EC mean scores classified participants as competent eaters at the beginning and after one year, with an increase in the internal regulation domain. Improvements in technical culinary knowledge were associated with gains in contextual skills. **Conclusions:** Participation in structured cooking classes positively influenced the development of CS and EC internal regulation among nutrition students.

## 1. Introduction

In recent decades, profound transformations in global dietary patterns have contributed to the increased prevalence of chronic non-communicable diseases, particularly those associated with the consumption of quick meals and unhealthy foods, as well as the decline of traditional cooking practices [1,2]. This context has prompted public health initiatives to encourage culinary education as a means to improve diet quality and food autonomy [3]. The ability to plan, select, prepare, and handle food safely—encompassed in the concept of culinary skills—is recognized as a determinant of healthier food choices and nutritional adequacy [4]. These characteristics are included in the Satter Eating Competence Model, which describes eating competence as a multidimensional framework encompassing attitudes toward food and eating, contextual skills for meal planning and management, internal regulation of hunger and satiety, and food acceptance [5]. EC has been previously evaluated across different populations [6,7,8,9,10,11,12,13,14,15,16,17], and studies indicate that individuals who are competent eaters tend to have better dietary quality and healthier eating behaviors [18].

The Food Guide for the Brazilian population [19] emphasizes the role of home cooking and the consumption of fresh foods in promoting health and preventing chronic diseases. However, despite growing awareness, young adults—especially university students—frequently report limited cooking skills, a lack of time, and a high dependence on ready-to-eat foods, which contribute to unhealthy dietary behaviors [20,21]. For nutrition students, this paradox is even more concerning as they are expected to be role models and health educators, yet may lack the practical experience needed to support and promote healthy eating. An exploratory and descriptive cross-sectional study, aimed to gain a deeper understanding of students’ perceptions of food and healthy diets in the fields of Human Nutrition and Dietetics and Food Science and Technology at the University of Barcelona, revealed that, despite their understanding of what a healthy diet entails, students encounter practical barriers, such as their family’s eating habits or a lack of free time, which can hinder their ability to apply theoretical knowledge to their daily routine [22]. In another study conducted with nutrition students in the United Kingdom during their final year of study, the intensity of internships, food insecurity, and social pressures experienced at the University were identified as factors that can make it challenging to consolidate healthy eating habits and develop skills aligned with nutrition training [23]. Aligned with this, a cross-sectional study of nutrition and dietetics interns in the United States found that exhaustion and the rushed nature of internships made it difficult for them to maintain a healthy diet [24]. This reinforces the difference between expectations and the reality of eating practices during training, suggesting that time constraints and food insecurity may influence healthy eating and culinary practices [24].

Culinary interventions in academic settings have shown promising results, particularly when structured with hands-on components and long-term follow-up [25,26]. In a randomized controlled trial among Brazilian university students, a cooking skills program significantly improved participants’ culinary confidence and dietary patterns, with effects sustained for 6 months [20]. Recent findings among Brazilian adults confirm that greater culinary and food skills are positively associated with higher eating competence [21]. In this sense, the hypothesis raised for this study was that participation in cooking classes improves the cooking skills and eating competence of nutrition students. Therefore, this study aimed to evaluate the impact of participating in cooking classes on the development of cooking skills and eating competence among nutrition students over 12 months.

## 2. Materials and Methods

### 2.1. Study Design and Settings

This is a longitudinal study of a sample of nutrition students from a public university in the Federal District, Brazil. Data collection took place from February 2022 to December 2024, during which participants were followed for 1 year.

All undergraduate students from the Nutrition course at the University who were enrolled in mandatory undergraduate subjects involving culinary practices were invited to participate in the study. These subjects are sequential, and each lasts 6 months, totaling 1 year (12 months) of contact with culinary practices. The first subject is offered to students in the fourth semester of the Nutrition course, and if they are approved for the first one, they are enrolled in the second subject.

Data were collected at the beginning of the first culinary practices university subject (“técnica dietética 1” and “técnica dietética 2”), at the end of the first subject (six months later), and at the end of the second subject (one year after the start of the first subject). Data collection occurred using Google Forms.

The culinary subjects consisted of weekly theoretical and practical cooking classes (4 h per class), featuring both technical (food preparation) and educational (meal planning, sustainability, food culture, etc.) content. The subjects aim to teach the technical procedures involved in food preparation; the physical and chemical changes that occur during culinary processes; and the culinary indices related to these changes and their importance in menu planning. Furthermore, these subjects provide knowledge of the culinary applications for each type of food and the nutritional transformations that result from the processes they undergo. Students receive guidance and are encouraged to develop healthy preparations and special preparations for individuals with dietary restrictions. In this way, it is expected that the student will be able to understand the relationship between nutritional value/cost/presentation of food in menu planning; identify and monitor the transformations and losses that food undergoes from the raw state to cooking; differentiate and apply various methods of pre-preparation and preparation of food; use food appropriately in different pre-preparation and preparation procedures; and prepare varied and healthy meals. Culinary subjects are offered in the 4th and 5th periods (midway through the 10-period course). At the end of each culinary subject, students were required to develop new recipes based on the culinary appointments related to ingredients, cooking techniques, and processes related to food restrictions. The focus of the final test is not on dietary restriction, but on applying knowledge of processes, techniques, and ingredients to assess learning during the period. The main variables studied were cooking skills (assessed by specific questionnaires) and eating competence (assessed using the ecSatter Inventory).

### 2.2. Participants

We used a convenience sample of students from the University’s undergraduate Nutrition course. Eligible criteria to participate in the study were (i) students from the University’s nutrition undergraduate course; (ii) agreement to participate in the survey and sign the consent form; (iii) enrollment in subjects that involved culinary practices; (iv) passing both subjects.

All students enrolled in the mentioned subjects from February 2022 to December 2024 were invited to participate in the study, totaling 137 potential participants. Those who did not meet the inclusion criteria, or those who did not complete the survey in the three specified periods (at the start of the first culinary practices subject, at the end of the first subject, and at the end of the second subject), were excluded from the sample.

### 2.3. Instruments

The instruments were composed of three parts: (i) sociodemographic data; (ii) Brazilian cooking skills and healthy eating questionnaire (QBHC) [27,28]; and (iii) Satter Eating Competence Inventory (ecSI2.0™BR), translated and validated for the Brazilian population [29,30,31].

A structured questionnaire captured sociodemographic variables, including age, gender, ethnicity, place of residence in Brazil, educational level, monthly household income, and the number of children and adults living in the household. Body mass index (BMI) was calculated [32].

Cooking skills were evaluated using the QBHC [27,28]. The instrument consists of 36 items, assessed on seven scales, and scores are obtained by averaging the coded responses for the items comprising each scale. The first part of the questionnaire consisted of an evaluation of the “Availability and Accessibility of Fruits and Vegetables Index” (AAFV). The score classification is as follows: 0–2 points—low availability of fruits and vegetables; 3–6 points—moderate availability of fruits and vegetables; 7–8 points—high availability of fruits and vegetables [28].

The second part of the questionnaire consisted of five topics evaluating Cooking Attitude (CA), Cooking Behavior (CB), Self-Efficacy in consuming Fruits, Vegetables, and Seasonings (SEFVS), Cooking Self-Efficacy (SEC), and Self-Efficacy in Using Fruits, Vegetables, and Seasonings (SEFVS) measures. The total score for the 2nd part (cooking skills) is classified as follows: 20–43 points—low cooking skills; 44–73—moderate cooking skills; and 74–100—high cooking skills [28].

The last part of the questionnaire evaluated “Knowledge in Cooking Terms and Techniques Evaluation” (CTT) [28]. The third part score classification is as follows: <6 points—low Knowledge in Cooking Terms and Techniques; ≥6 points—high Knowledge in Cooking Terms and Techniques [28].

Eating competence was assessed through the Brazilian version of the Satter Eating Competence Inventory (ecSI2.0™BR) (translated and validated for the Brazilian population) [29,30]. The ecSI2.0™BR is available upon request on the NEEDs Center website and requires authorization for use. The ecSI2.0™BR is composed of 16 items scored on a five-point Likert scale (Always = 3 to Rarely/Never = 0). The score is obtained by summing the responses for each item. Therefore, the total score varies from 0 to 48 [33]. To be considered a competent eater, the participant must score at least 32, and higher scores indicate greater EC [4,5]. The instrument, composed of the three aforementioned parts, was inserted into the Google Forms platform for data collection.

### 2.4. Data Collection

Recruitment and data collection occurred from February 2022 to December 2024. The data collection process occurred in three periods: (i) in the 1st class of the subject that involved culinary practices; (ii) in the last class of the first subject that involved culinary practices (six months later); (iii) in the last class of the second subject that involved culinary practices (12 months postbaseline). The invitation was reinforced through WhatsApp groups. The link containing the instrument was sent to all students enrolled in Nutrition courses that involved culinary practices.

Before starting the survey, participants were provided with an informed consent form that explained the study’s purpose, potential risks, and the confidentiality of their responses. Only those who agreed to participate by selecting the option “I have read and accept the Informed Consent Form” proceeded to respond to the questionnaire.

### 2.5. Statistical Analysis

Descriptive statistics of the sociodemographic characteristics were presented using means and standard deviations for continuous variables and frequencies and percentages for categorical variables. Scores from the Cooking Skill Questionnaire (CSQ) and ecSI2.0™BR are expressed as means and standard deviations. Comparisons of CSQ and ecSI2.0™BR scores across the three assessment times were conducted using one-way repeated measures ANOVA, followed by Bonferroni’s post hoc test. The relationships between CSQ and ecSI2.0™BR scores, including their changes over time, were evaluated using Pearson’s correlation coefficient. The normality of the data was assessed using the Shapiro–Wilk test. All statistical tests were two-tailed with a significance level of 0.05. Statistical analyses were performed using SPSS^®^ software version 20.0.

### 2.6. Ethical Aspects

This study was conducted in accordance with the Declaration of Helsinki and the Research Ethics Committees (approval number: 3769157), and was approved by the NEEDs Center, holder of the copyright for the tool that assesses eating competence (ecSI2.0™BR).

## 3. Results

**Figure 1:**
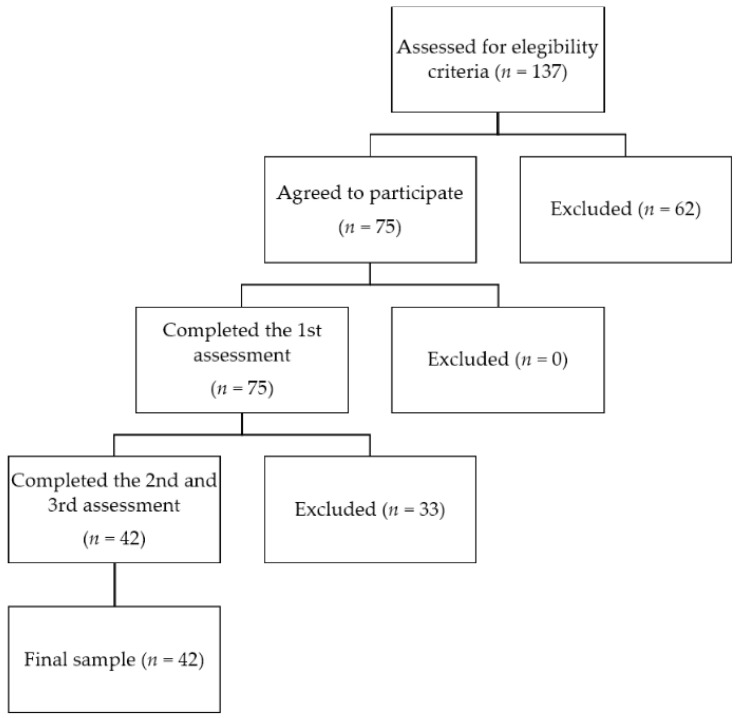
Flowchart of recruitment, exclusions, and loss to follow-up.

Of the 137 potential participants (students enrolled in subjects involving culinary practices from February 2022 to December 2024), 75 agreed to participate and completed the 1st assessment. Twenty-seven completed only the 1st assessment, and six completed only the 3rd assessment. Therefore, 33 were excluded from the final sample because they did not meet the inclusion criteria of completing the entire instrument in all three assessment periods (Figure 1). The age, sex, and BMI of the participants excluded from the study did not differ significantly (*p* > 0.05) from those of the study sample (Table S1). A total of 42 met all inclusion criteria and completed the entire instrument across the three periods, forming the study sample. Participants (*n* = 42) were predominantly female (*n* = 33; 78.6%) and aged 19–35 years (mean = 21.07 ± 2.71 years). Over the course of one year, the BMI mean increased among participants (*p* = 0.029) (Table 1).

The EC mean total score for the last period (12 months from the start of subjects involving culinary practices) was ≥32 [5]. The mean total score did not statistically differ among the three periods (*p* > 0.05) (Table 1). At baseline, 59.5% of participants (*n* = 25) were considered competent eaters (EC ≥ 32). In the 2nd and 3rd assessments, despite an increase in the mean EC score, 24 participants were considered competent eaters (57.1% of the sample). Internal regulation was the only EC domain that increased after one year of subjects involving culinary practices.

According to the classification adopted for the QBHC, the “Availability and Accessibility of Fruits and Vegetables Index (AAFV)” was considered moderate (7.0–8.0) from the beginning for participants (mean ranging from 6.64 to 6.99) [28]. Participants were also classified as having high cooking skills (74–100) (part 2) from the beginning [16]. However, the scores were higher at 6 and 12 months after the start of the subject’s involvement in culinary practices (*p* < 0.05). Considering the “Knowledge in Cooking Terms and Techniques Evaluation” (CTT), at the 1st data collection, the mean classification showed participants as having low “Knowledge in Cooking Terms and Techniques” (<6 points) and after the 2nd and 3rd data collection (e.g., at the end of the 1st semester of cooking classes and the end of the 2nd semester of cooking classes), the mean classified them as high Knowledge in Cooking Terms and Techniques (≥6 points) [28]. Despite this, no significant difference was found when comparing the three periods.

Before participants started subjects involving culinary practices (1st data collection) and six months later (2nd collection), a positive correlation was found between total, food acceptance, and contextual skills EC scores and the cooking skills QBHC score (Table 2). After 12 months of involvement in culinary practice subjects, a positive correlation was found between all EC domains and the cooking skills QBHC score. Regarding the QBHC score, which evaluates the accessibility of fruits and vegetables, only EC contextual skills were positively correlated in the first data collection. QBHC Knowledge in Cooking Terms and Techniques was not associated with any EC domains or the total.

Table 3 presents the correlations of the evolution of scores over time to answer the following question: “Those who, over time (completion of the subjects), increased their EC the most, also increased their QBHC scores?”). Those who increased EC contextual skills (1st and 2nd data collection—after 6 months) also increased QBHC Knowledge in Cooking Terms and Techniques (0.358), indicating that individuals who improved their contextual skills over time also enhanced their knowledge in cooking techniques. Those who increased their EC eating attitudes (between the 1st and 3rd data collection—after 12 months) also increased their QBHC cooking skills (r = 0.37) and improved their Self-Efficacy in consuming Fruits, Vegetables, and Seasonings. No significant associations were observed between the evolution of eating competence and culinary skills during the transition from the 2nd to the 3rd data collection.

## 4. Discussion

This is the first study to evaluate the impact of participating in cooking classes on the development of cooking skills and eating competence among nutrition students over one year. As expected, most participants were females (*n* = 33; 78.6%) and young (mean age of 21.07 ± 2.71), since it is well reported in the literature that females generally participate more in research involving health, and they are also the ones who are most involved in culinary practices [34,35,36,37,38]. In addition, in Brazilian public universities, there is a predominance of young adults in undergraduate courses [39].

Participants reached the cutoff for competent eaters (EC ≥ 32) only at the final evaluation (12 months), with no significant difference in total EC scores across the three data collection points, despite their mean EC score being <32 in the first two evaluations. However, an increase was observed in the internal regulation domain, consistent with findings from a Brazilian study that analyzed the relationship between cooking and food skills and eating competence, showing that higher levels of these abilities are associated with better eating competence, particularly in the domain of internal regulation [21]. Our findings align with data showing that eating competence, with a focus on internal regulation, is linked to motivation and planning skills [40]. A study in the UK with almost 80% of undergraduate students, mostly female (68.9%) and younger than 25 years (41.1%), showed that healthcare course students were more knowledgeable about good nutritional practices, which include knowledge and interest in nutrition, food choices, and food handling [41]. Among Brazilian college students, a previous study reported that health science students had greater EC than exact and earth science students [42], corroborating our results.

A Brazilian study evaluating a culinary program for university students found improvements in the availability and accessibility of fruits and vegetables at home after the intervention. These improvements were maintained even six months after the intervention [20]. In the present study, the availability and accessibility of fruits and vegetables were considered moderate from the beginning. This highlights the importance of the home food environment in promoting healthy eating habits among university students. A Dutch study examining the interaction between the food environment and cooking skills, as well as their association with diet-related outcomes in adults, suggests that even when a person possesses good cooking skills, the external food environment can negatively affect dietary outcomes [43]. Although it was not directly evaluated, this can be potentially related to the increase in BMI observed after 1 year among the students who participated in our study [43]. Nevertheless, it is important to emphasize that, in subjects related to cooking at the University of this study, in addition to tasting what is produced during classes, students have, as a final project, the development of foods for special purposes (food-related disorders or unusual dietary patterns). They test several recipes outside the classroom environment (in the cooking laboratory or at home) and taste them until they achieve the desired final product. Although it is recommended to taste products in small portions, this is not controlled, and people may consume large portions, potentially affecting energy intake and leading to a consequent increase in body weight, which can affect BMI.

The findings of the present study about Knowledge in Cooking Terms and Techniques (CTT) are consistent with another study demonstrating the effectiveness of culinary interventions in this regard [20]. In the current study, the CTT mean was initially classified as low, but after 1 year, it had significantly increased to a mean of ≥6 points, considered high [28]. This is consistent with what the aforementioned Brazilian study, a randomized controlled trial with 78 students, also demonstrated: after the intervention, there was an increase in CTT knowledge and its long-term maintenance [20]. This similarity reinforces the idea that culinary programs can enhance cooking knowledge and improve familiarity with cooking terms and techniques, even among a group that already has a good understanding of the field.

The participants’ EC was positively correlated with their overall cooking skills, before and after a year of culinary classes. Before the culinary subjects, only the contextual skills and food acceptance domains showed a positive correlation with overall cooking skills, and contextual skills were only correlated with the accessibility and availability of fruits and vegetables. Knowledge in Cooking Terms and Techniques was not associated with any EC domains. These results can be partially related to a study that explored the relationship between cooking and food skills and eating competence among Brazilian adults, and with a study that correlated EC to executive function skills in college students, which observed a significant positive correlation between cooking skills and EC, emphasizing strong links with total EC, food acceptance, and contextual skills [21,40]. Confidence in using fruits and vegetables is associated with positive eating attitudes, as observed in a study of Brazilian university students. The same study indicates that contextual skills can support better organization of access to healthy food [44].

Previous Brazilian publications have reported an inverse association of EC and BMI [30,42]. However, in the present study, the average BMI increased among participants over the course of 1 year, but EC did not differ. A study with a sample of 1720 American college students investigated which factors were more predictive of EC, the BMI, or the attitude about their weight, and the results revealed that weight satisfaction and desire to lose weight were better predictors of EC than BMI [8]. Considering that weight gain is multifactorial, many things may have changed in the individual’s life, contributing to the observed increase in BMI. Therefore, in the present study, it is not possible to attribute a direct relationship between BMI and EC. In addition, although we observed some improvements after the cooking classes, we did not compare participants to a control group of nutrition students who did not take these specific classes. Therefore, it may be attributed to cooking classes or participants’ maturation during the academic year, which may characterize a potential limitation of the study.

## 5. Conclusions

This study demonstrated that participation in cooking practice courses over one year contributed to improvements in culinary skills. Although students already exhibited high levels of eating competence and culinary skills at baseline, relevant improvements were observed, particularly in the internal regulation domain of eating competence and in technical knowledge of cooking terms and techniques. The results revealed positive correlations between the development of culinary skills and various dimensions of eating competence, reinforcing the value of practical and sustained interventions in academic settings as educational strategies. Therefore, it is recommended that cooking practice courses be maintained and strengthened within nutrition programs, with encouragement for continued longitudinal monitoring of their effects and expansion of such initiatives to other health-related fields. Even with a small sample of students, the study shows that cooking practice courses should be encouraged in nutrition programs. Nutritionists who acquire cooking techniques and culinary skills may help the population in a different way to achieve a healthier diet.

## Figures and Tables

**Table 1:** Comparison of participants’ BMI, eating competence, and cooking skills scores over the three evaluation periods (interval of 1 semester between each measure) *.

		1st Data Collection	2nd Data Collection	3rd Data Collection	*p* **
	**BMI**	23.5 (4.2) ^a^	23.6 (4.4) ^ab^	23.81 (4.45) ^b^	0.029
	**Eating competence**				
	Eating attitudes	12.5 (3.3) ^a^	12.2 (3.9) ^a^	12.5 (3.9) ^a^	0.703
	Food acceptance	5.6 (2.3) ^a^	5.6 (2.2) ^a^	5.8 (2.1) ^a^	0.604
	Internal regulation	3.9 (1.4) ^a^	3.7 (1.6) ^a^	4.2 (1.5) ^b^	0.046
	Contextual skills	9.8 (2.9) ^a^	9.6 (2.6) ^a^	9.6 (2.8) ^a^	0.838
	TOTAL	31.7 (6.9) ^a^	31.1 (7.7) ^a^	32.1 (7.7) ^a^	0.417
	ecSI2.0™BR ≥ 32	25 (59.5%) ^a^	24 (57.1%) ^a^	24 (57.1%) ^a^	0.922
	**Brazilian cooking skills and healthy eating questionnaire (QBHC)**				
Part 1: Accessibility of Fruits and Vegetables	Availability and Accessibility of Fruits and Vegetables Index (AAFV)	6.9 (1.4) ^a^	6.6 (1.5) ^a^	7.0 (1.6) ^a^	0.336
Part 2: Cooking skills	Cooking Attitude (CA)	14.8 (3.0) ^a^	14.2 (2.9) ^a^	14.0 (2.6) ^a^	0.115
	Cooking Behavior (CB)	9.5 (2.3) ^a^	9.9 (2.6) ^a^	9.9 (2.6) ^a^	0.454
	Self-Efficacy in Produce Consumption (SEPC)	9.7 (2.8) ^a^	10.3 (2.6) ^a^	10.0 (3.5) ^a^	0.349
	Cooking Self-Efficacy (SEC)	23.6 (3.8) ^a^	25.2 (3.4) ^b^	25.7 (3.0) ^b^	<0.001
	Self-Efficacy in Using Fruits, Vegetables, and Seasonings (SEFVS)	15.9 (3.2) ^a^	16.3 (2.8) ^a^	16.3 (2.6) ^a^	0.588
	TOTAL–cooking skills	73.4 (1.6) ^a^	75.9 (1.5) ^b^	75.95 (1.5) ^b^	0.034
Part 3: Knowledge in Cooking Terms and Techniques	Knowledge in Cooking Terms and Techniques Evaluation (CTT)	5.9 (1.5) ^a^	6.3 (1.2) ^a^	6.55 (1.0) ^a^	0.065

* Data were collected at the beginning of the first culinary practices university subject, at the end of the first subject (six months later), and at the end of the second subject (one year after the start of the first subject). ** One-way repeated-measure ANOVA with Bonferroni’s post hoc tests. Periods with the same letters do not differ significantly (*p* > 0.05).

**Table 2:** Pearson correlation coefficients between eating competence (EC) and Brazilian cooking skills and healthy eating questionnaire (QBHC) scores in the three assessment periods ^a^.

QBHC		1st Data Collection				
		EC				
		Eating Attitudes	Food Acceptance	Internal Regulation	Contextual Skills	TOTAL
Part 1: Accessibility of Fruits and Vegetables	AAFV (Part 1)	0.060	0.150	0.065	0.350 *	0.234
Part 2: Cooking skills	CA	0.212	0.122	0.119	0.250	0.267
	CB	0.024	0.264	−0.286	0.200	0.124
	SEPC	0.180	0.565 *	0.051	0.521 *	0.495 *
	SEC	0.131	0.435 *	−0.158	0.359 *	0.322 *
	SEFVS	0.242	0.391 *	−0.023	0.413 *	0.408 *
	Total (Part 2)	0.242	0.533 *	−0.080	0.523 *	0.489 *
Part 3: Knowledge in Cooking Terms and Techniques	CTT (Part 3)	−0.134	0.094	−0.026	0.129	0.015
**QBHC**		**2nd Data Collection**				
		**EC**				
		**Eating** **Attitudes**	**Food** **Acceptance**	**Internal** **Regulation**	**Contextual Skills**	**TOTAL**
Part 1: Accessibility of Fruits and Vegetables	AAFV (Part 1)	−0.002	0.150	−0.166	0.285	0.105
Part 2: Cooking skills	CA	0.266	0.267	0.270	0.179	0.327 *
	CB	−0.130	0.213	−0.220	0.005	−0.048
	SEPC	0.323 *	0.438 *	0.227	0.583 *	0.533 *
	SEC	0.293	0.433 *	0.176	0.446 *	0.460 *
	SEFVS	0.260	0.404 *	0.217	0.314 *	0.399 *
	Total (Part 2)	0.312 *	0.527 *	0.209	0.462 *	0.508 *
Part 3: Knowledge in Cooking Terms and Techniques	CTT (Part 3)	0.025	0.098	0.046	0.228	0.128
**QBHC**		**3rd Data Collection**				
		**EC**				
		**Eating** **Attitudes**	**Food** **Acceptance**	**Internal** **Regulation**	**Contextual Skills**	**TOTAL**
Part 1: Accessibility of Fruits and Vegetables	AAFV (Part 1)	0.134	0.063	0.133	0.037	0.125
Part 2: Cooking skills	CA	0.260	0.207	0.172	0.312 *	0.335 *
	CB	−0.080	0.326 *	−0.060	0.113	0.077
	SEPC	0.461 *	0.338 *	0.442 *	0.558 *	0.614 *
	SEC	0.238	0.315 *	0.395 *	0.495 *	0.461 *
	SEFVS	0.360 *	0.256	0.338 *	0.494 *	0.496 *
	Total (Part 2)	0.385 *	0.432 *	0.403 *	0.602 *	0.609 *
Part 3: Knowledge in Cooking Terms and Techniques	CTT (Part 3)	0.106	−0.083	0.244	0.154	0.133

^a^ Data were collected at the beginning of the first culinary practices university subject, at the end of the first subject (six months later), and at the end of the second subject (one year after the start of the first subject). * Statistically significant (*p* < 0.05). AAFV—Availability and Accessibility of Fruits and Vegetables Index; CA—Cooking Attitude; CB—Cooking Behavior; SEPC—Self-Efficacy in Produce Consumption; SEC—Cooking Self-Efficacy; SEFVS—Self-Efficacy in consuming Fruits, Vegetables, and Seasoning; CTT—Knowledge in Cooking Terms and Techniques Evaluation.

**Table 3:** Pearson correlation coefficients between the evolution of eating competence scores and culinary skills in the three evaluation periods.

QBHC		1st–2nd Data Collections				
		EC				
		Eating Attitudes	Food Acceptance	Internal Regulation	Contextual Skills	TOTAL
Part 1: Accessibility of Fruits and Vegetables	AAFV (Part 1)	−0.190	−0.003	−0.284	0.067	−0.153
Part 2: Cooking skills	CA	0.155	0.131	0.199	0.220	0.249
	CB	−0.022	0.008	−0.192	−0.082	−0.088
	SEPC	0.165	0.062	0.032	0.259	0.198
	SEC	0.117	−0.076	−0.025	−0.088	−0.002
	SEFVS	0.178	0.022	0.051	−0.056	0.093
	Total (Part 2)	0.220	0.043	−0.007	0.068	0.146
Part 3: Knowledge in Cooking Terms and Techniques	CTT (Part 3)	0.207	0.081	0.033	0.358 *	0.258
**QBHC**		**1st–3rd Data Collections**				
		**EC**				
		**Eating** **Attitudes**	**Food** **Acceptance**	**Internal** **Regulation**	**Contextual Skills**	**TOTAL**
Part 1: Accessibility of Fruits and Vegetables	AAFV (Part 1)	−0.086	−0.036	0.053	−0.125	−0.095
Part 2: Cooking skills	CA	0.180	0.081	0.008	0.089	0.168
	CB	0.028	0.068	−0.109	0.017	0.016
	SEPC	0.365 *	−0.074	0.169	0.128	0.288
	SEC	0.192	0.008	−0.283	−0.091	0.011
	SEFVS	0.325 *	−0.114	0.044	0.121	0.216
	Total (Part 2)	0.373 *	−0.015	−0.046	0.094	0.242
Part 3: Knowledge in Cooking Terms and Techniques	CTT (Part 3)	0.059	0.020	0.071	−0.003	0.059
**QBHC**		**2nd–3rd Data Collections**				
		**EC**				
		**Eating** **Attitudes**	**Food** **Acceptance**	**Internal** **Regulation**	**Contextual Skills**	**TOTAL**
Part 1: Accessibility of Fruits and Vegetables	AAFV (Part 1)	−0.084	−0.125	0.029	−0.137	−0.135
Part 2: Cooking skills	CA	0.044	0.115	0.164	−0.145	0.050
	CB	−0.048	0.181	−0.270	0.000	−0.050
	SEPC	0.229	−0.192	0.050	0.117	0.134
	SEC	0.285	−0.077	0.070	0.162	0.225
	SEFVS	0.043	−0.034	−0.079	−0.091	−0.046
	Total (Part 2)	0.228	0.008	−0.017	0.019	0.136
Part 3: Knowledge in Cooking Terms and Techniques	CTT (Part 3)	0.098	−0.019	0.057	0.009	0.071

* Statistically significant (*p* < 0.05). AAFV—Availability and Accessibility of Fruits and Vegetables Index; CA—Cooking Attitude; CB—Cooking Behavior; SEPC—Self-Efficacy in Produce Consumption; SEC—Cooking Self-Efficacy; SEFVS—Self-Efficacy in consuming Fruits, Vegetables, and Seasoning; CTT—Knowledge in Cooking Terms and Techniques Evaluation.

## Data Availability

The original contributions presented in this study are included in the article. Further inquiries can be directed to the corresponding authors.

## Supplementary Materials

The following supporting information can be downloaded at: https://www.mdpi.com/article/10.3390/nu18020259/s1, Table S1. Comparison of the characteristics of participants who completed and those who did not complete the study.

## Abbreviations

The following abbreviations are used in this manuscript:

AAFV	Availability and Accessibility of Fruits and Vegetables Index
BMI	Body mass index
CA	Cooking attitude
CB	Cooking behavior
CS	Cooking skills
CSQ	Cooking Skills Questionnaire
CTT	Knowledge in Cooking Terms and Techniques Evaluation
EC	Eating competence
SEC	Cooking self-efficacy
SEFVS	Self-efficacy in consuming fruits, vegetables, and seasonings
