# Supplementary material for: Do Cooking Classes for Nutrition Students Improve Their Eating Competence and Cooking Skills? A 1-Year Follow-Up in a Sample of Brazilian Public University Students
Source: Nutrients. 2026 Jan 14;18(2):259. doi: 10.3390/nu18020259 (PMC12845400; doi:10.3390/nu18020259)
Supplement: Supplementary file 1 [file nutrients-18-00259-s001.zip › nutrients-4070762-supplementary.pdf]

**Table S1.** Comparison of the characteristics of participants who completed and those who did not complete the study.

| | Completed the study n=42 | Did not complete the study n = 33 | p |
|---|---|---|---|
| **Age (mean; SD)** | 21.07 (2.71) | 22.12 (2.48) | 0.087* |
| **Sex** | | | |
| Female | 33 (78.6%) | 20 (60.6%) | 0.090** |
| Male | 9 (21.4%) | 13 (39.4%) | |
| **BMI (mean; SD)** | 23.5 (4.2)[a] | 22.9 (3.85) | 0.576* |

* Student t-test

** Pearson chi-squared test
